# HIV Testing among Patients with Presumptive Tuberculosis: How Do We Implement in a Routine Programmatic Setting? Results of a Large Operational Research from India

**DOI:** 10.1371/journal.pone.0156487

**Published:** 2016-05-31

**Authors:** Ajay MV Kumar, Devesh Gupta, Ashok Kumar, R. S. Gupta, Avinash Kanchar, Raghuram Rao, Suresh Shastri, MD Suryakanth, Chethana Rangaraju, Balaji Naik, Deepak K. Guddemane, Prashant Bhat, Achuthan Sreenivas Nair, Anthony David Harries, Puneet Dewan

**Affiliations:** 1 International Union Against Tuberculosis and Lung Disease (The Union), South-East Asia Regional Office, New Delhi, India; 2 Central TB Division, Directorate General of Health Services, Ministry of Health and Family Welfare, Government of India, New Delhi, India; 3 National AIDS Control Organization, Ministry of Health and Family Welfare, Government of India, New Delhi, India; 4 World Health Organization, Geneva, Switzerland; 5 State TB Cell, Directorate of Health Services, Bangalore, Karnataka, India; 6 Karnataka State AIDS prevention and control Society, Bangalore, Karnataka, India; 7 World Health Organization Country Office for India, New Delhi, India; 8 International Union Against Tuberculosis and Lung Disease (The Union), Paris, France; 9 London School of Hygiene and Tropical Medicine, London, United Kingdom; 10 Bill & Melinda Gates Foundation, New Delhi, India; University of Malaya, MALAYSIA

## Abstract

**Background:**

In March 2012, World Health Organization recommended that HIV testing should be offered to all patients with presumptive TB (previously called TB suspects). How this is best implemented and monitored in routine health care settings in India was not known. An operational research was conducted in Karnataka State (South India, population 64 million, accounts for 10% of India’s HIV burden), to test processes and learn results and challenges of screening presumptive TB patients for HIV within routine health care settings.

**Methods:**

In this cross-sectional study conducted between January-March 2012, all presumptive TB patients attending public sector sputum microscopy centres state-wide were offered HIV testing by the laboratory technician, and referred to the nearest public sector HIV counselling and testing services, usually within the same facility. The HIV status of the patients was recorded in the routine TB laboratory form and TB laboratory register. The laboratory register was compiled to obtain the number of presumptive TB patients whose HIV status was ascertained, and the number found HIV positive. Aggregate data on reasons for non-testing were compiled at district level.

**Results:**

Overall, 115,308 patients with presumptive TB were examined for sputum smear microscopy at 645 microscopy centres state-wide. Of these, HIV status was ascertained for 62,847(55%) among whom 7,559(12%) were HIV-positive, and of these, 3,034(40%) were newly diagnosed. Reasons for non-testing were reported for 37,700(72%) of the 52,461 patients without HIV testing; non-availability of testing services at site of sputum collection was cited by health staff in 54% of respondents. Only 4% of patients opted out of HIV testing.

**Conclusion:**

Offering HIV testing routinely to presumptive TB patients detected large numbers of previously-undetected instances of HIV infection. Several operational challenges were noted which provide useful lessons for improving uptake of HIV testing in this important group.

## Introduction

According to the World Health Organization (WHO) Global TB Report 2014, an estimated 1.1 million people globally had HIV-associated tuberculosis (TB), and 360,000 died from HIV-associated TB in 2013. [1] Three important reasons have been given for this unacceptably high death rate: i) in persons with HIV/AIDS, TB was not diagnosed and treated; ii) in patients with TB, HIV was not diagnosed and thus co-infected patients were not referred to HIV care and treatment; and iii) when the two diseases were diagnosed and treated, this often happened far too late to be effective.[2]

In March 2012, WHO launched its updated policy on collaborative TB/HIV activities to reduce the burden of TB and HIV. This policy uses the same framework as the 2004 WHO Policy of TB/HIV collaborative activities, but includes some important new recommendations, one of which is that routine HIV testing and counselling should be offered not only to patients diagnosed with TB, but also to those with presumptive TB (previously called TB suspects).[3] This recommendation was based on the findings from studies done predominantly in sub-Saharan African countries, which show a high HIV prevalence among patients with presumptive TB ranging from 27%-64%, sometimes even higher than HIV prevalence among TB patients.[4–10] However, these findings from HIV endemic settings were not generalizable to India, a country with a ‘concentrated’ HIV epidemic (meaning HIV prevalence in general population remains lower than 1%).[11] Hence, despite recommendations from WHO, routine HIV testing had been offered only to ‘TB patients’ and not to all ‘patients with presumptive TB’ in India. Operational research conducted in two districts of South India in 2010 showed that the HIV prevalence among presumptive TB patients can be as high as that among TB patients ranging from 7%-10%, and that ‘Provider initiated HIV testing and counselling’ (PITC, a term used when health care providers actively offer the HIV testing to certain patient groups) among presumptive TB patients can be feasibly implemented in settings with decentralized HIV testing facilities with potential for increased HIV case finding, early treatment initiation and reduction in mortality and morbidity.[12, 13]

The National Technical Working Group (NTWG) for joint TBHIV collaborative activities in India acknowledged the strong evidence and took a policy decision to implement PITC among presumptive TB patients in high HIV settings in India.[14] However, how this is best implemented and monitored in routine health care settings, especially when implemented at large scale, was not known, and this implementation-knowledge gap needed to be addressed. The roles and responsibilities of the different staff officers were unclear, and mechanisms for recording and reporting in routine settings had not been tested. So, NTWG recommended the pilot implementation of PITC in one State of India for a period of 3–6 months to finalize the operational guidance before scale-up to other high HIV settings.[14] Accordingly, a pilot study was implemented in the entire state of Karnataka (South India, population 64 million, high HIV prevalence). Karnataka State was chosen for two main reasons: 1) Large state enabling to test operational feasibility of the strategy when PITC is implemented to scale 2) relatively high HIV prevalence and better TB-HIV related health infrastructure compared to other states in India. Duration of three months was chosen for the pilot so as to understand the operational challenges.

This paper describes the screening procedures deployed (including mechanisms for recording, reporting and monitoring), the experience of implementation, results and challenges of screening presumptive TB patients for HIV within routine health care settings of Karnataka State, India.

The specific objectives were to assess

1. the proportion of patients with presumptive TB whose HIV status was ascertained, disaggregated district-wise
2. the proportion HIV positive among those who had their HIV status ascertained, disaggregated district-wise
3. the reasons for not ascertaining the HIV status, from the perspective of the health care-provider
4. the number of HIV cases “newly” diagnosed and the number eligible for antiretroviral therapy (ART eligibility was assessed using WHO-2013 ART guidelines [all TB patients irrespective of CD4 count and for presumptive TB patients without TB, a CD4 count <500/mm3 was considered ART eligible] in line with the recent decision of national AIDS control organisation in India) [15, 16]
5. The number needed to screen (NNS) to find an additional case of HIV-infection among “patients with presumptive TB” in comparison to “confirmed TB patients” and those in reproductive age group (25–54 years).
6. The average increase in daily workload at the HIV testing centers as a result of using this strategy (obtained by dividing the total number of Presumptive TB patients who underwent HIV testing by average number of working days in each centre)

## Materials and Methods

### Ethics approval

Approval of the competent national (Central TB Division and National AIDS Control Organization) and state authorities (State TB Cell and Karnataka State AIDS prevention and control society) was obtained for conducting this pilot. Ethics approval was obtained from Institutional Ethics Committee of the National Tuberculosis Institute, Bangalore, India. Individual written informed consent from each patient was not taken separately for the study as this was intended to be a pilot programme implementation and standard operating procedures of National AIDS Control Programme (NACP) were followed for counselling and HIV testing, which included obtaining written consent. The same was approved by the ethics committee including waiver of a separate informed consent. Given the programmatic nature of the study and availability of local ethics approval, the Ethics Advisory Group of International Union Against Tuberculosis and Lung Disease waived the need for ethics review. Data was maintained securely by programme staff and electronic databases contained no personal identifiers.

### Design

This was a cross-sectional, implementation research study conducted among patients with presumptive TB examined for diagnostic smear microscopy within the routine health services of the state of Karnataka, India.

### Setting

Karnataka has an estimated 0.21 million people living with HIV in 2011 and accounts for about 10% of country’s HIV burden.[11] Hence, the state has been classified as ‘high priority’ for HIV interventions by the National AIDS Control Organization (NACO) in India on the basis of consistently high HIV sero-prevalence rates of >1% among antenatal women and >5% in other high risk groups. [17, 18] In the state, tuberculosis control programme services are available through a decentralized network of peripheral health institutions which provide general health services including diagnosis and treatment for TB.

Patients with presumptive TB (defined as people with cough for two weeks or more with or without other symptoms suggestive of TB) are identified at the peripheral health centres and referred for sputum smear microscopy to Designated Microscopy Centres (DMCs), which are geographically distributed, each covering a population of 0.05 to 0.1 million. In situations where the patient is unable to physically visit the DMC, the sputum is collected and transported by the health care workers in the general health system or non-governmental organizations. The diagnosis of TB is made in accordance with national guidelines, and all diagnosed TB patients are treated with standardized fully intermittent thrice-weekly short-course regimens (6–9 months) administered under direct observation. Such patients are registered at one of the 125 sub-district level TB programme management units (which are administrative and supervisory units for every 10–15 peripheral health institutions) according to Indian programme guidelines. [19]

As per national policy, HIV status is routinely ascertained for TB patients, and HIV-infected TB patients are provided Cotrimoxazole Preventive Therapy and referred to Anti-Retroviral Treatment (ART) centres for initiation on ART. [20] TB patients are referred for free HIV counselling and testing to one of the 1637 ‘integrated counselling and HIV testing centres’ (ICTC, these are peripheral health institutions with HIV testing services) throughout the state. At the time of the study, about 90% of DMCs had co-located HIV testing services. Free ART is provided through a network of 44 ART centres (with at-least one ART centre in every district), where HIV-infected patients (including TB patients) are started on treatment and care. Once patients were clinically stable on ART, they were referred to one of 121 Link-ART centres in the state, closer to the patient’s residence for continuing on ART. These service delivery sites under NACP follow the national guidelines for counselling, testing, care and treatment of HIV-infected patients. [21] In line with the higher burden of HIV, there is a higher density of HIV testing and care centres in the northern parts of the state. [22]

### Study population

All patients with presumptive TB examined for diagnostic smear microscopy at the DMCs of Karnataka state from January-March 2012 formed the study population.

### Sample size and Sampling

Since this was a pilot study intended to be implemented under programmatic conditions to answer questions of feasibility, it was decided to enrol all patients with presumptive TB (including children aged 0–14 years) examined for smear microscopy during the pilot period in all the 31 districts of the state. No patients were excluded. For this reason, sample size calculations were deemed not relevant for the study.

### Planning and implementation

Following the decision of NTWG and based on the official government orders from the Revised National Tuberculosis Control Programme (RNTCP) and NACP, joint-meetings were organized between the state level programme managers for planning and rolling out the activities, training of the staff and ensuring supply chain management of HIV test kits.

All staff involved in data collection, data validation and data entry were trained in carrying out the respective procedures. The District TB Officers, District nodal officer of HIV/AIDS and WHO Consultants of the respective study district were trained at state level as master trainers who then trained the district level staff and oversaw the smooth implementation of study. The trainings were conducted jointly for the implementing staff (TB field supervisors, Laboratory technicians of DMCs and HIV testing centres, Counsellors of HIV testing centres) by programme managers of the state level with support from WHO consultants.

Regular supervisory visits were conducted by the state level programme managers (once a month), WHO Consultants (once a fortnight) and district programme managers (once a fortnight). Periodic review meetings were conducted to assess the progress of the pilot and address any implementation issues.

**Fig 1:**
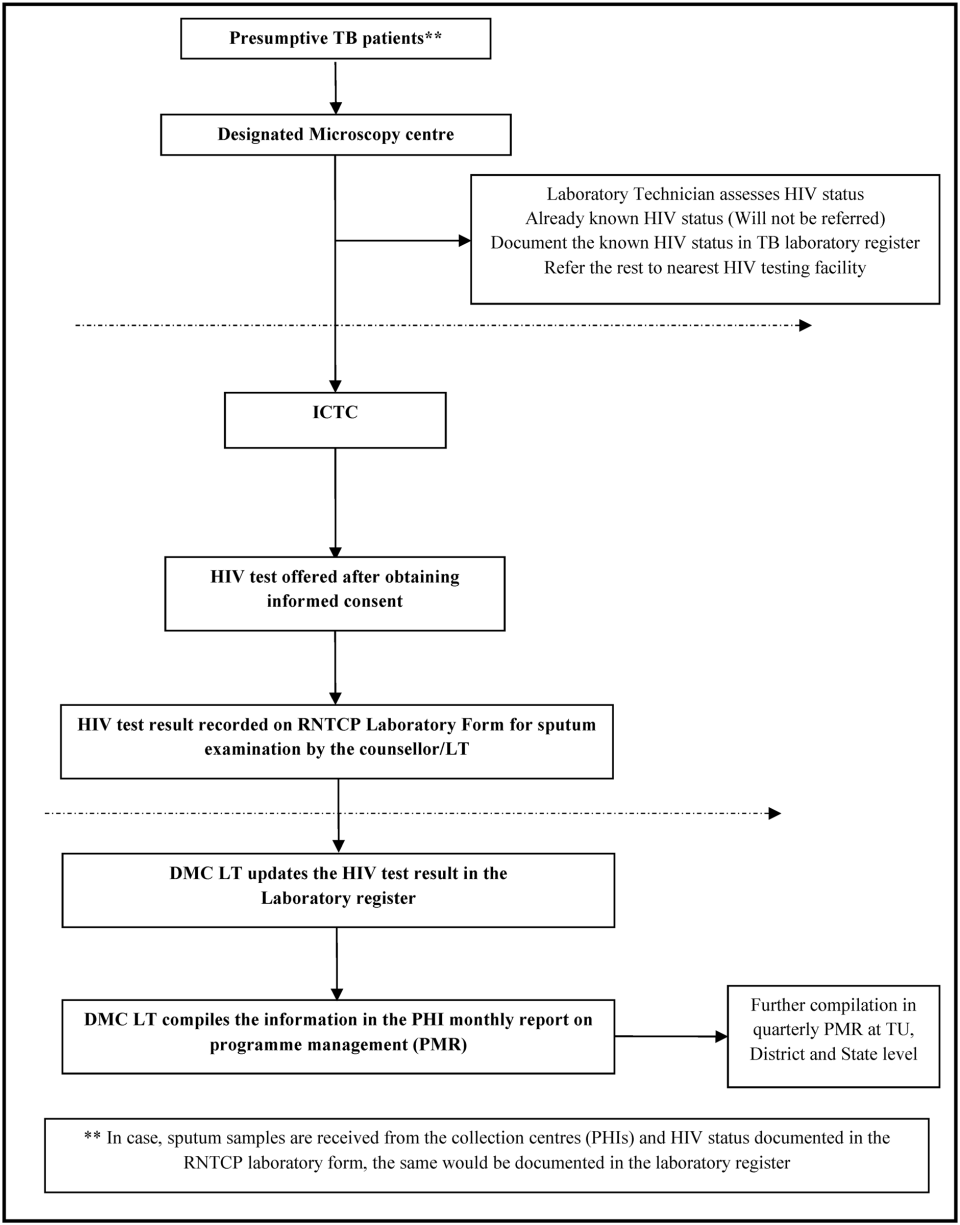
Flow Chart depicting the patient flow and the recording and reporting mechanism, PITC among presumptive TB patients, Karnataka, India, 2012. PITC-Provider initiated HIV testing and counselling; HIV-Human immunodeficiency virus; TB-tuberculosis; RNTCP-Revised National Tuberculosis Control Programme; PHI-Peripheral Health Institutions; TU-Tuberculosis Unit; LT-Laboratory Technician; DMC-Designated Microscopy Centre; PMR-Programme Management Report.

**Fig 2:**
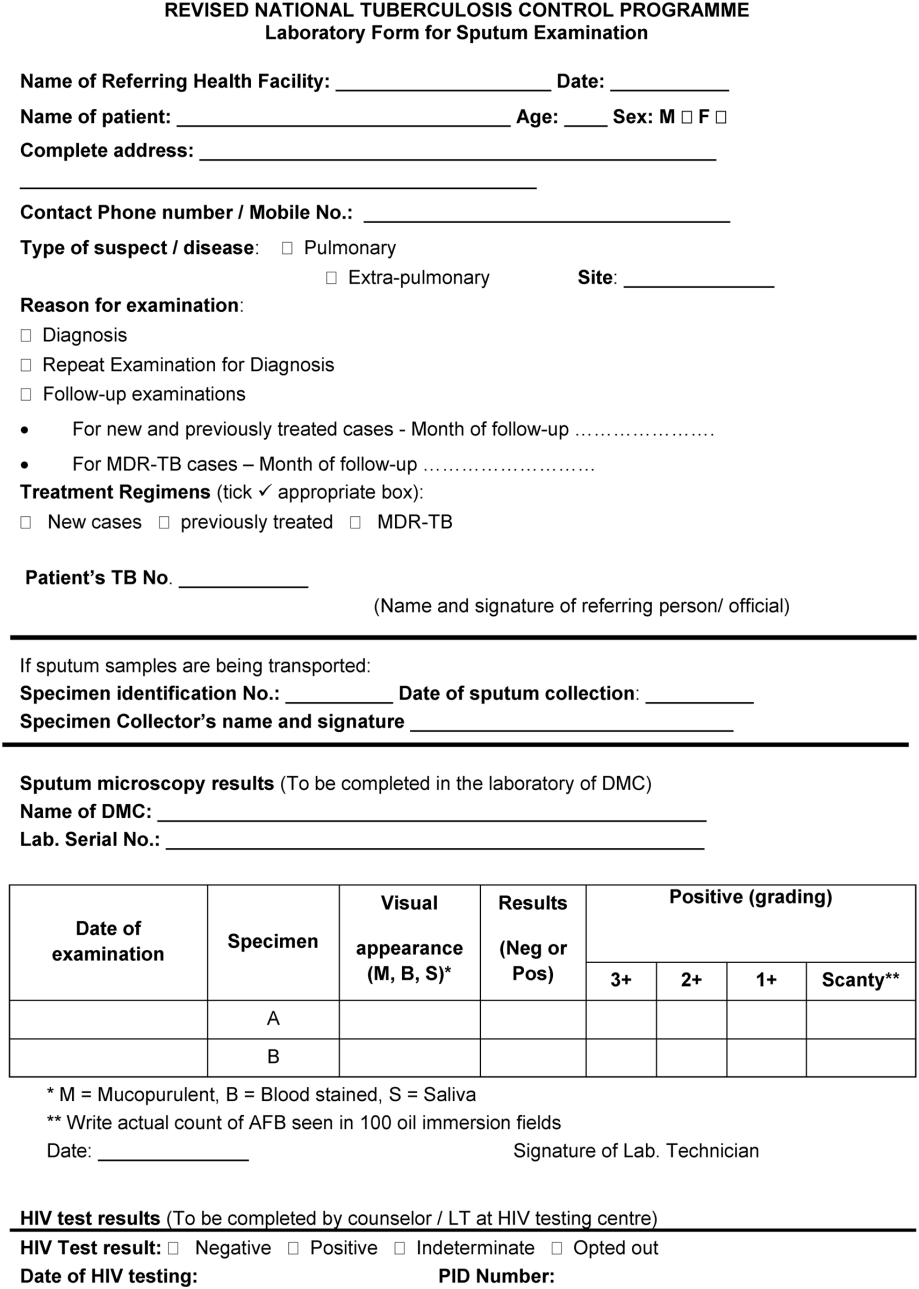
Modified format of laboratory form for sputum examination, PITC among presumptive TB patients, Karnataka, India, 2012.

**Fig 3:**
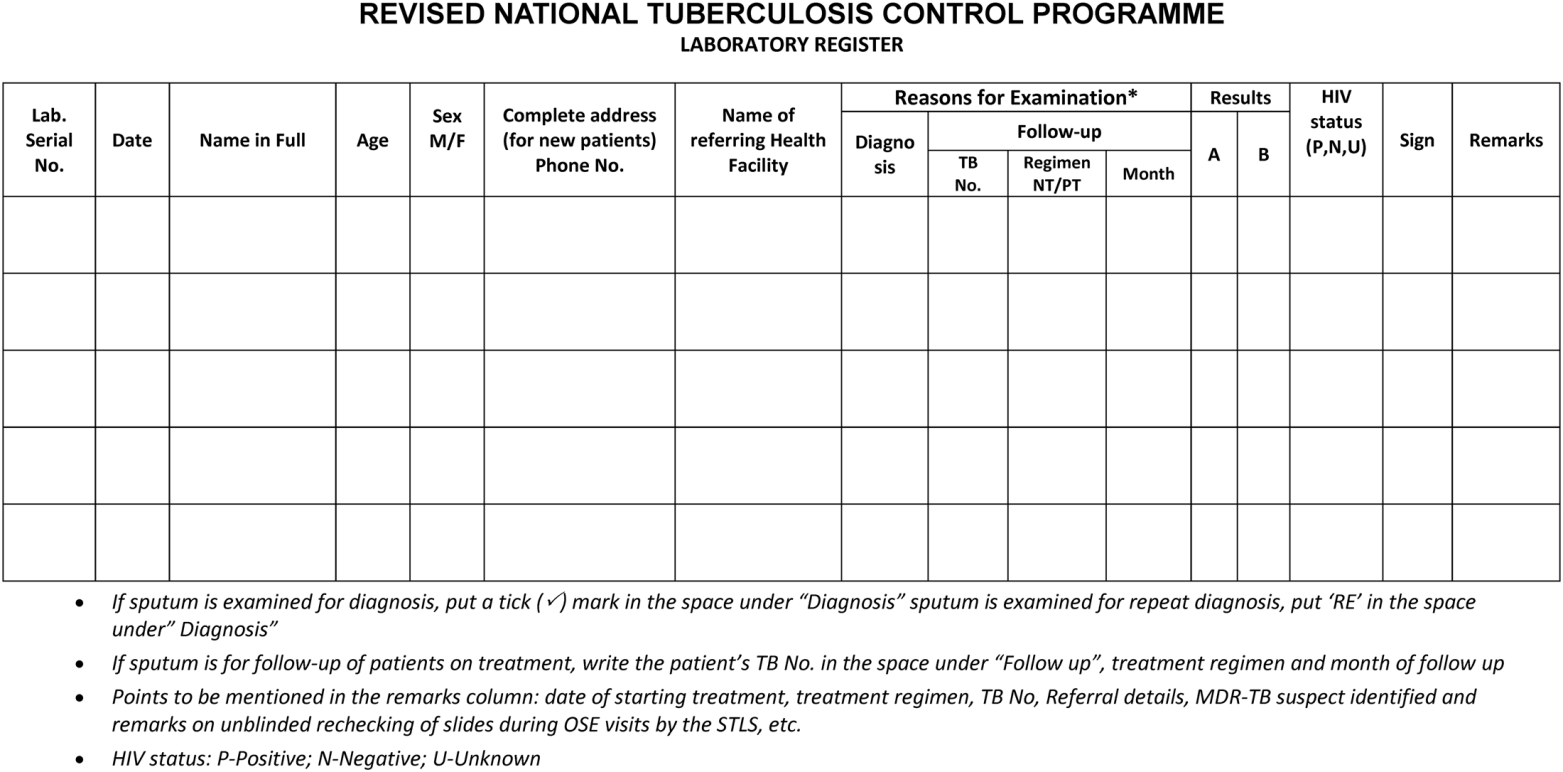
Modified format of laboratory register, PITC among presumptive TB patients, Karnataka, India, 2012.

The screening procedures and the patient flow in the health system are shown in Figs 1–3 and described in Table 1.

**Table 1:** Operational procedures in provider-initiated HIV counselling and testing among patients with presumptive TB in Karnataka state, India, January-March 2012.

Operational Procedure	Steps
**Procedure at Designated Microscopy Centre (DMC)**	**Step 1**: All Presumptive TB patients (including those coming for a repeat sputum examination) attending the designated microscopy centre (DMC) for diagnosis of TB were offered HIV counselling and testing by the DMC laboratory technician (DMC LT).
	**Step 2**: DMC LT after receiving the first sputum sample, referred the patient to the nearest, preferably the co-located HIV testing facility. If the patient was known to be HIV positive (in case of referral from ART centres, ICTCs or blood banks), the information regarding the HIV status was recorded in the laboratory register and the patient was not referred any further. The date of HIV testing was extracted from the ART patient booklet; if not available, the approximate date of HIV testing was documented as per patient’s history. The information regarding **‘HIV status’ and ‘date of HIV testing’** was documented in the **RNTCP laboratory register** (in a new column added for the purpose). In the case of presumptive TB patients whose HIV status was not known or HIV negative, LT referred them to the nearest or co-located HIV testing facility.
	**Step 3**: In cases where only the sputum samples reached the DMC (in the absence of the patient), it was recorded in the remarks column of the laboratory register. No active effort to track and offer an HIV test to such patients was undertaken. However, if there was a HIV testing facility at the peripheral health facilities (as in some districts like Bagalkot and Belgaum), HIV testing was offered to all presumptive TB patients whose sputum was collected at the collection points. The information regarding HIV status and date of HIV testing was then collected by DMC LT from the collection centres and this was updated in the laboratory register.
	**Step 4**: The counsellor at the HIV testing centre provided the feedback regarding HIV status (Positive/Negative/Indeterminate/Opted out) and date of HIV testing in the RNTCP laboratory form for sputum examination. A new field to capture HIV status and date of HIV testing was created in the RNTCP laboratory form for sputum examination. DMC LT updated the laboratory register based on the feedback on the laboratory form. Alternatively, the DMC LT discussed with the counsellor at the end of the day and updated the HIV status in the laboratory register—this is as per the national guidelines of shared confidentiality among health care providers for providing optimum care for the patient.
	**Step 5**: The predominant reason for not ascertaining the HIV status, wherever known, was recorded in the remarks column.
**Procedure at the Integrated Counselling and HIV Testing Centre (ICTC)**	**Step 1**: Presumptive TB patients coming to the ICTCs were offered counselling and testing as per the norms and standard operating procedures of the NACP.
	**Step 2**: All referrals were recorded in the ICTC counselling register as referrals from RNTCP.
	**Step 3**: Presumptive TB patients who were known to be HIV positive and patients who had tested HIV negative within last 6 months were not re-tested. Patients who had an indeterminate result were re-offered the HIV test.
	**Step 4**: For patients with HIV positive results, the counsellor linked these patients to the nearest ART centre available in the district/state. This was done by giving a referral form and explaining to the patient about how to access the centre. The patient was given the contact details of the district programme managers for any assistance.
	**Step 5**: The counsellor then documented the HIV status in the RNTCP laboratory form as feedback to DMC LT. The counsellor also assisted the DMC LT to update the laboratory register with information on HIV status.
**Recording**	**RNTCP laboratory form for sputum examination** (Fig 2): A new field was created to capture the HIV status and date of HIV testing which was completed by the counsellor/LT at the HIV testing centre wherein one of the following options was noted—Positive, Negative, Indeterminate, Opted out.
	**RNTCP laboratory register** (Fig 3): A column to capture HIV status was added in the laboratory register (Positive, Negative, Unknown)
**Reporting**	The following indicators were added in the monthly report submitted by each DMC to RNTCP: (1) Of Presumptive TB patients examined for diagnosis, number with known HIV status. (2) Of above, number HIV positive. This information was then compiled quarterly in the district and State level programme management reports and reported in routine RNTCP surveillance.

Since this was a pilot study and the objective was also to study the processes, separate data collection forms were used to capture individual patient information which included information on presence or absence of a co-located ICTC, age, sex, sputum smear result, HIV status, prior HIV status and CD4 count. These variables were extracted into a pre-tested structured data collection format by the Senior TB laboratory supervisor (STLS) in co-ordination with the LT and the counsellor at the HIV testing centre. STLS then coordinated with the district TB/HIV supervisor and referred to the HIV patient records maintained at the ART centre to obtain information on the baseline CD4 count. Aggregate data on reasons for non-testing were extracted from the remarks column of the laboratory register and compiled at district and state level. Once the pilot period (January-March 2012) was over, the separate data collection formats were discontinued.

### Data Validation

The STLSs were trained in extracting data from the laboratory register accurately and expected to visit the DMCs under their purview once a week to ensure that activities were implemented as per protocol including recording of HIV status in the laboratory register. This was in turn monitored by District TB Officer and the WHO field consultant once in a fortnight.

### Data entry & Analysis

In this multi-centre study, we used a method of coordinating data capture by utilizing a combination of three open access sources (EpiData for data entry, Dropbox for sharing data and TeamViewer for trouble-shooting remotely). [23] All data entry was done by the district-level data entry operators working for RNTCP. The technique has been detailed elsewhere.[23] Double data entry, validation and analysis were done using EpiData software (version 3.1 for entry and version 2.2.2.182 for analysis, EpiData Association, Odense, Denmark). Given the large numbers in the study, it is expected to find statistical significance even when the actual differences between groups are small. Hence, we have refrained from showing results of any statistical tests and make all the interpretations from programmatic point of view.

## Results

Data was obtained from all the 31 districts in the state. This included 645 microscopy centres, among which 573(89%) had HIV testing services available in the same facility. A total of 115,308 patients with presumptive TB [40% female, mean age of 44 years] were examined for sputum smear microscopy. HIV status was ascertained for 62,847 (55%) among whom 7,559 (12%) were found to be HIV positive.

**Fig 4:**
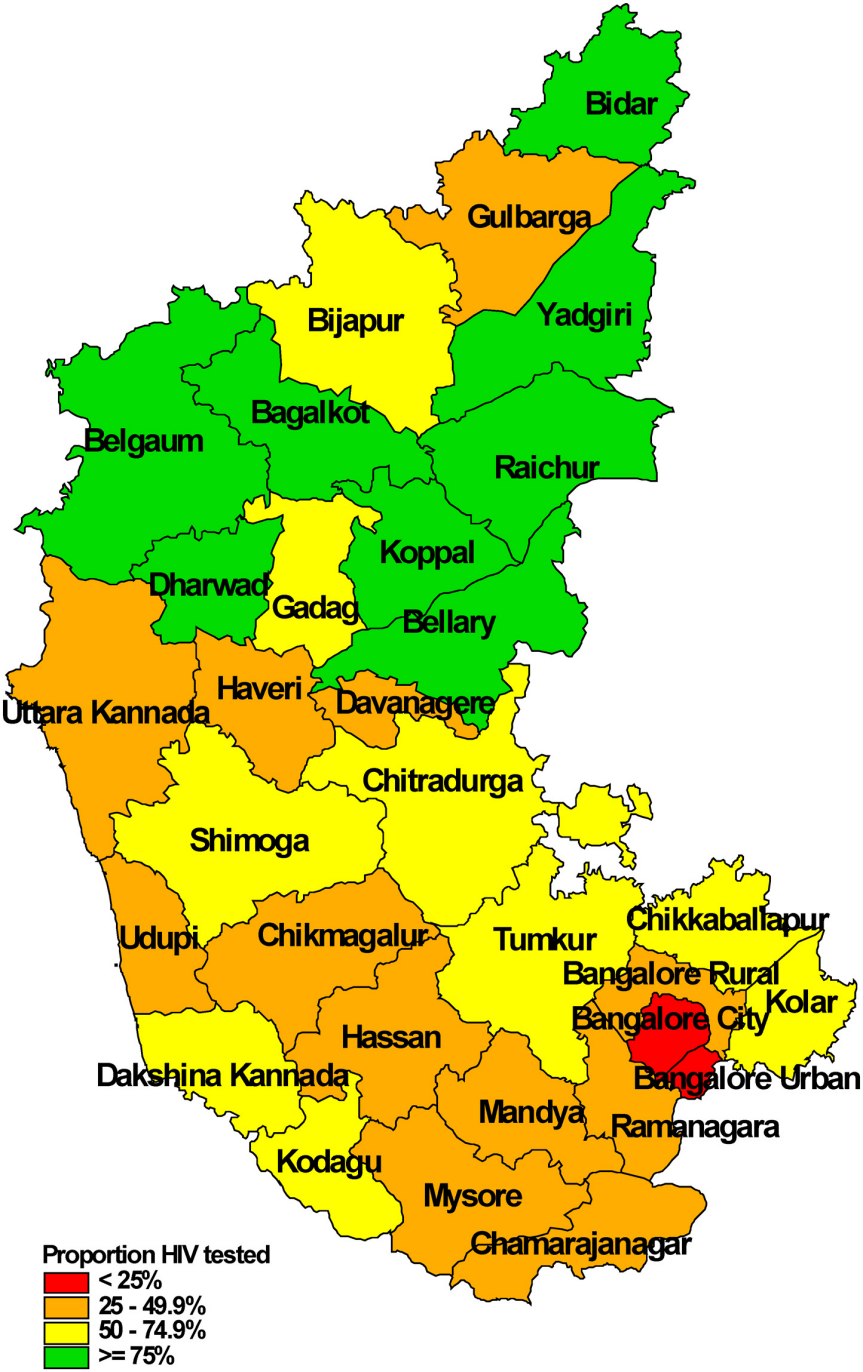
Ascertainment of HIV status among patients with presumptive TB, district-wise, in Karnataka state, India, January-March 2012.

The proportion whose HIV status was ascertained along with HIV positive results, disaggregated by age, sex and smear-positivity, is shown in Table 2. HIV testing varied little [ranged between 50% and 57%] across age groups and sexes. HIV testing rates were two times higher among patients visiting microscopy centres with co-located HIV testing facilities as compared to those without. Patients with confirmed smear-positive TB were more likely to be tested for HIV as compared to those with other types of TB. HIV testing rates varied between 15% and 95% across districts with districts in the northern part of the state performing better as compared to southern part (Table 3, Fig 4).

**Table 2:** Ascertainment of HIV status and HIV positivity among patients with presumptive TB in Karnataka state, India, January-March 2012.

Parameter	Number examined for sputum smear microscopy	Number (%) with HIV status ascertained	Number (%) HIV Positive
**Total**	115308	62847 (55)	7559 (12)
**Age (years)**			
00–14	3808	1887 (50)	252 (13)
15–24	12804	7326 (57)	514 (7)
25–34	19595	11276 (57)	2157 (19)
35–44	20707	11738 (57)	2580 (22)
45–54	20336	11080 (55)	1333 (12)
55–64	18808	10022 (53)	510 (5)
≥65	18857	9448 (50)	205 (2)
Unknown	393	70 (18)	8 (11)
**Sex**			
Male	69189	37498 (54)	4088 (11)
Female	46068	25335 (55)	3465 (14)
Transgender	7	6 (86)	5 (83)
Unknown	44	8 (18)	1 (13)
**Sputum Smear status**			
Smear Positive	9789	7071 (72)	802 (11)
Smear Negative	105519	55776 (53)	6757 (12)
**HIV testing facility**			
Not co-located	6248	1784 (29)	114 (6)
Co-located	109060	61063 (56)	7445 (12)

TB—Tuberculosis; HIV—Human immunodeficiency virus;

**Table 3:** Ascertainment of HIV status and HIV positivity among patients with presumptive TB, district-wise, in Karnataka state, India, January-March 2012.

Parameter	Number examined for sputum smear microscopy	Number (%) with HIV status ascertained	Number (%) HIV Positive
**Bagalkot**	3351	3021 (90)	812 (27)
**Bangalore City**	9524	2330 (25)	251 (11)
**Bangalore Rural**	2054	591 (29)	39 (7)
**Bangalore Urban**	5311	816 (15)	61 (8)
**Belgaum**	8569	8122 (95)	959 (12)
**Bellary**	3533	2827 (80)	380 (13)
**Bidar**	2987	2540 (85)	137 (5)
**Bijapur**	2801	1873 (67)	530 (28)
**Chamarajanagar**	2735	899 (33)	117 (13)
**Chikkaballapur**	2523	1307 (52)	91 (7)
**Chikmagalur**	2928	1169 (40)	84 (7)
**Chitradurga**	2365	1613 (68)	71 (4)
**Dakshina Kannada**	4116	2761 (67)	170 (6)
**Davanagere**	3156	1070 (34)	250 (23)
**Dharwad**	3899	3261 (84)	451 (14)
**Gadag**	2237	1321 (59)	216 (16)
**Gulbarga**	4348	1835 (42)	193 (11)
**Hassan**	4938	2374 (48)	156 (7)
**Haveri**	2223	877 (40)	113 (13)
**Kodagu**	1317	661 (50)	33 (5)
**Kolar**	2733	1841 (67)	283 (15)
**Koppal**	2435	2032 (83)	446 (22)
**Mandya**	5026	2327 (46)	187 (8)
**Mysore**	6962	2407 (35)	180 (8)
**Raichur**	3201	2595 (81)	444 (17)
**Ramanagara**	3052	1463 (48)	38 (3)
**Shimoga**	3576	1788 (50)	209 (12)
**Tumkur**	5955	3391 (57)	405 (12)
**Udupi**	2455	1187 (48)	84 (7)
**Uttara Kannada**	3783	1573 (42)	97 (6)
**Yadgiri**	1215	975 (80)	72 (7)

**Fig 5:**
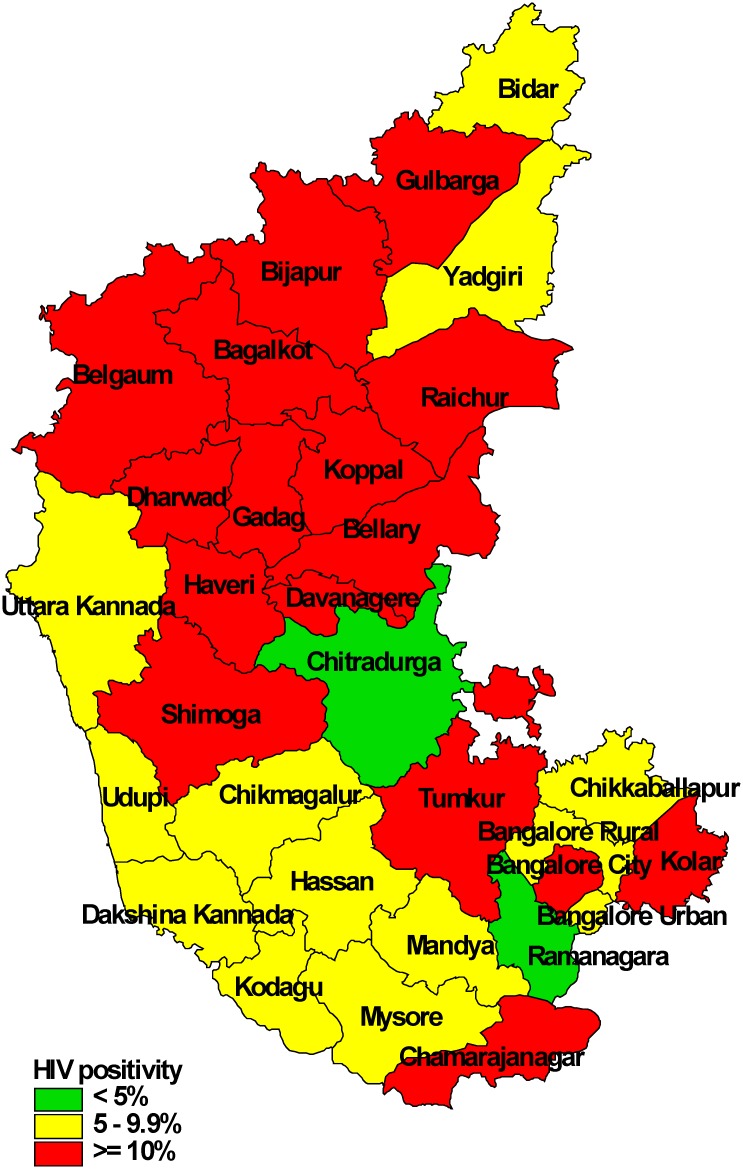
HIV positivity among patients with presumptive TB, district-wise, in Karnataka state, India, January-March 2012.

HIV positivity tended to be lower among elderly age groups, marginally higher among females as compared to males and varied between 3% and 28% across districts—with a higher positivity in districts located in the northern part of the state. HIV positivity was similar among smear-positive and smear-negative patients (Table 3, Fig 5).

Of all presumptive TB patients in the study, 4525 were already diagnosed as HIV positive prior to the study. Of the remaining 110783 patients with unknown status prior to the study, HIV status could be ascertained for 53627 (48%) patients, in whom an additional 3034 HIV positive patients were identified. Thus, of a total of 7,559 HIV-positive patients identified, 3,034 (40%) were newly diagnosed with HIV infection as a result of this screening effort. Among 3,034 newly diagnosed HIV cases, ART eligibility could be assessed for 2,244 (74%) of whom 1,992 (89%) had CD4 count ≤500 and were found to be eligible for ART initiation. (Table 4)

**Table 4:** Antiretroviral therapy (ART) eligibility in newly diagnosed HIV among patients with presumptive TB, Karnataka state, India, January-March 2012.

Characteristic	TB patients	Presumptive TB patients without TB	Total
**Number of newly diagnosed HIV cases**	395	2639	3034
**Number (%) assessed for ART eligibility**	395 (100)	1849 (70)	2244 (74)
**Number (%) ART eligible**	395 (100)	1597 (87)	1992 (89)
**Median (IQR) CD4 count**	149 (74–290)	195 (89–361)	189 (87–355)

When hypothetically comparing potential policies for HIV testing, the option of limiting HIV testing to adults in 25–54 years was most efficient. In this analysis, we excluded patients with previously known HIV status and examined the yield of HIV among those with a previously unknown HIV status. (Table 5)

**Table 5:** Number needed to screen (NNS) to find one new (previously undiagnosed) case of HIV, by hypothetical strategy, Karnataka state, India, January-March 2012.

Strategy	Number with previously unknown HIV status tested for HIV	Number (%) HIV Positive	NNS
**HIV testing for all smear-positive TB patients**	6035	395 (6.5)	15
**HIV testing for all presumptive TB patients**	53627	3034 (5.7)	18
**HIV testing of all presumptive TB patients in the age group 25–54 years**	27769	2408 (8.7)	11

The reasons for non-ascertainment of HIV status, collected from 37,300 (72%) of the 52,461 patients without HIV testing, is shown in Table 6. The key reason for non-testing was related to the fact that sputum samples were collected at peripheral health institutions and transported to microscopy centres instead of referring patients.

**Table 6:** Reasons for non-ascertainment of HIV status among presumptive TB patients, Karnataka state, India, January-March 2012.

Reasons for non-ascertainment of HIV status	Number	Percentage
Sputum specimens reaching microscopy centre instead of presumptive TB patients	20,454	54%
Non-availability of HIV test kits	7,179	19%
Non-availability of staff (Laboratory Technician, Counsellor) at the time of referral (due to leave, outreach duty, travel to attend meetings)	3,681	10%
HIV testing facility not co-located at microscopy centres	2,332	6%
Opted out of HIV testing	1,659	4%
Other reasons (lack of awareness among staff, gaps in recording, refusal to test due to workload, non-referral due to misconceptions of the staffs, death)	2,395	7%
**Total**	37,700*	**100%**

* Data about reasons for non-testing was available from 37,300 (72%) of the 52,461 patients

A workload assessment indicated that the median [inter quartile range] increase in the number of clients to be tested for HIV per day as a result of this intervention was 2 [1–2]. About 90% of the HIV testing centres had an increase of less than or equal to five clients per day (Table 7).

**Table 7:** Increase in workload at HIV testing centres due to strategy of ‘routine HIV testing of presumptive TB patients’, Karnataka state, India, January-March 2012.

Average increase in number of clients tested for HIV	Number (%) [based on actual numbers tested for HIV]	Number (%) [assuming all TB suspects will be tested for HIV]
1–2 clients per day	396 (69.5)	252 (44.2)
3–5 clients per day	141 (24.7)	224 (39.3)
6–10 clients per day	31 (5.4)	79 (13.9)
>10 clients per day	2 (0.4)	15 (2.6)

HIV-Human immunodeficiency virus;

## Discussion

The study showed that the strategy of HIV testing among patients with presumptive TB could be feasibly and effectively implemented within the routine health system. We consider the intervention was feasible for the following reasons: 1) All the interventions including recording and reporting were implemented in a large state by the existing staff and resources. One of the major strengths of this study is that it was done with complete engagement of the national programmes from the stage of planning to execution, monitoring, recording and reporting. The existing programme resources were used for implementing all the activities without the need for any extra budget. There was a total increase in the need for HIV test kits which in turn needed optimisation of supply chain management and we need to factor-in this aspect in future procurement cycles. 2) There was minimal additional workload in the HIV testing centres. Barring a small proportion of tertiary care institutes with high patient load, the average daily increase in number of patients requiring to be tested was low at more than 95% of HIV testing centres. In centres which had an increase of more than 10 clients tested per day, additional staff may need to be deployed. 3) Only 4% of the patients opted out of HIV testing indicating high acceptability of this intervention among the patient population.

We consider the strategy to be effective as it led to finding thousands of new HIV-positive patients who were ART eligible. Nearly 40% of the 7559 HIV-positive cases were ‘newly diagnosed’ as a result of this strategy with almost 90% of them eligible for ART as per the existing criteria for starting ART. Nearly half of all presumptive TB patients were tested for HIV, although district-wise analysis indicated large variability. This is probably due to variation in availability of HIV testing facilities and their co-location at the sputum microscopy centres.

There are several important points that merit discussion. First, while WHO now recommends routine HIV testing of all presumptive TB patients, the operational guidance as to how to do this is lacking. This is among the first studies to fill that gap and addresses an area of global priority. This was also a national priority and addressed a specific request from the NTWG of Government of India.[14] Previous studies on this issue have focussed on assessing prevalence of HIV among presumptive TB patients rather than testing implementation models in routine programme settings. [4–10] These findings were presented back to NTWG and a decision to nationally scale up this intervention across all high HIV settings was made. Changes in recording and reporting have already been adopted by the national TB and HIV programmes in India. While we cannot attribute it directly, we note that some of these changes are now reflected in global guidance. [24]

Second, this is one of the largest studies on the issue of HIV testing among presumptive TB patients with more than 0.1 million patients screened in a period of three months across one of the HIV priority states of India. Being carried out in the routine health care setting, this study gives valuable information of the ground realities and challenges in implementing a new strategy within the general health system.

Third, we found that nearly 90% of HIV-infected presumptive TB patients (without TB) were eligible for ART. This is similar to the findings of two recent studies from Zimbabwe and India.[25, 26] As a public health approach, this is another subset of patients (similar to HIV-infected persons having active TB disease, hepatitis B virus infection with severe chronic liver disease, being pregnant or breast feeding, being aged under five years, and living in a sero-discordant relationship) in whom ART should be recommended irrespective of CD4 count. This strengthens the argument about moving towards a HIV ‘test and treat’ strategy for presumptive TB patients. This is in line with the recent guidance from WHO which calls for starting all HIV patients on ART irrespective of CD4 count.[27]

Fourth, about half of the presumptive TB patients were not tested for HIV. Not surprisingly, HIV testing was more likely in DMCs with co-located HIV testing facilities. This justifies the ongoing efforts of the national programmes to prioritise setting up of new HIV testing facilities at hospitals having sputum microscopy facilities. This, however, will not address the major reason for non-testing in a substantial proportion of presumptive TB patients in whom sputum samples were collected and transported from peripheral health institutions to DMCs—such patients will miss the opportunity to get HIV tested at DMC. This calls for further decentralization of the HIV testing services to all the peripheral health institutions and sputum collection centres and national programmes should seriously consider this strategy to improve the uptake of HIV testing. We suggest scaling-up the use of rapid HIV screening tests at all the PHIs and those who are screened positive may be referred for further testing and confirmation at the ICTCs. The other programmatic challenge is to improve the procurement and supply chain management of HIV test kits to enhance HIV test uptake. Unavailability of laboratory technician or counsellors at DMC and/or ICTC was another reason for lack of HIV testing. This can be addressed by training the other staff within the general health system and motivating them to perform HIV testing in line with the overall approach to integrate NACP into the general health system and this would be sustainable in the long term.

Fifth, we captured data in an innovative manner using the freely available resources for data entry, storage, sharing and trouble shooting. This approach to ensuring quality of data capture in multicentre operational research in resource-constrained settings has been a great lesson.[23]

There were some limitations to our study given the operational nature of the study and reliance on routinely collected data. In this pilot, we could not capture the data among smear negative presumptive TB patients as to how many were eventually diagnosed with smear negative pulmonary TB and extrapulmonary TB. In assessing ART eligibility, information on whether the person was living in a sero-discordant relationship would have been useful, but was not captured. Including these criteria would have increased the number of people eligible for ART and hence the numbers obtained in this study are an underestimate. Furthermore, CD4 cell count data were missing in 30% of the HIV positive patients. Information on how many newly diagnosed HIV infected presumptive TB patients were put on ART and Co-trimoxazole Prophylactic Therapy later was also not documented. Information such as this would be very useful to assess if this strategy indeed increased the overall number of HIV-infected persons placed on ART and if it reduced the delay in initiating ART. Further studies are required to assess this and the impact on reducing overall HIV-TB associated mortality. While there could be multiple reasons in an individual for non-testing of HIV, only one predominant reason was captured and that too from the provider’s perspective. In about one-third of patients, reason for non-testing was not mentioned and this is a serious limitation. Further, we do not have any information if this sub-group is similar to the rest in whom we know the reason for non-testing. Future research involving qualitative methods should be undertaken to understand patients’ perspectives for not getting tested for HIV.

WHO and Joint United Nations Programme on HIV/AIDS (UNAIDS) have both embraced a bold new vision of “90-90-90” strategy which emphasizes the need to detect 90% of all HIV infected patients in the community, treat 90% of those detected with antiretroviral therapy (ART) and achieve viral suppression in 90% of those treated, and this has been reinforced by a similar 90-90-90 vision for TB control from the global STOP TB partnership.[28, 29] Achieving these ambitious targets will require innovative efforts to find previously undetected HIV cases in the community. Testing presumptive TB patients in Karnataka proved to be an efficient means of HIV case-finding, and may complement that effort.

In conclusion, this operational research demonstrated a feasible way to make HIV testing among presumptive TB patients a reality in routine health settings. Several operational challenges were noted which provide useful lessons for moving forward.
